# Diagnostic performance comparison of two ultrasound classification systems for hepatic alveolar echinococcosis: a single-center retrospective study

**DOI:** 10.1186/s40249-025-01402-9

**Published:** 2026-01-19

**Authors:** Yifei Wang, Jiaojiao Zhou, Wu Zhou, Xiaofei Zhong, Yongzhong Li, Diming Cai

**Affiliations:** Department of Medical Ultrasound, West China Hospital, Sichuan University, Chengdu, 610041 Sichuan Province China

**Keywords:** Hepatic alveolar echinococcosis, Ultrasound classification, Diagnostic performance, *Echinococcus multilocularis* Ulm Classification-Ultrasound, *Echinococcus multilocularis* National Health Commission Classification-Ultrasound

## Abstract

**Background:**

Hepatic alveolar echinococcosis (HAE) is a severe zoonotic parasitic disease for which ultrasonography is the primary diagnostic tool. However, the heterogeneous imaging characteristics of HAE lesions present significant challenges to accurate diagnosis. To improve diagnostic reliability, this study compared the performance of two established ultrasound classification systems: the *Echinococcus multilocularis* National Health Commission Classification-Ultrasound (EMNHCC-US) and the *E. multilocularis* Ulm Classification-Ultrasound (EMUC-US).

**Results:**

This study compared EMUC-US and EMNHCC-US systems in 169 HAE cases (179 lesions) and 99 non-HAE controls. Both systems identified heterogeneous echotexture as a universal feature but differed in lesion categorization and diagnostic performance. Inter-observer agreement was moderate for EMUC-US (κ = 0.57) and substantial for EMNHCC-US (κ = 0.73). The EMUC-US system included atypical patterns such as metastasis-like, found in 10.6% of cases, and ossified lesions, found in 6.1%. This contributed to its high sensitivity of 96.7% and a negative predictive value (NPV) of 90.3%. The EMNHCC-US system focused on advanced patterns, identifying infiltration in 49.7% of cases and liquefactive necrosis in 31.8%. It demonstrated superior specificity of 94.2% and a positive predictive value of 95.5%. Receiver operating characteristic analysis confirmed a better overall discriminative ability for EMNHCC-US, with an area under curve of 0.88 compared to 0.72 for EMUC-US. Sensitivity analysis revealed that EMUC-US maintained a near-perfect NPV of approximately 100% across all prevalence levels. In contrast, EMNHCC-US offered a higher PPV in high-prevalence settings.

**Conclusions:**

The EMUC-US and EMNHCC-US systems share core features, but EMUC-US includes smaller, atypical lesions (e.g., ossification, metastasis-like), enhancing early HAE detection with higher sensitivity and NPV, ideal for screening. EMNHCC-US focuses on advanced lesions, offering high specificity for confirmation. They are complementary: EMUC-US for sensitive screening, EMNHCC-US for specific diagnosis. Together, they enable a stratified strategy, optimizing case identification and clinical decisions despite lesion heterogeneity.

**Graphical Abstract:**

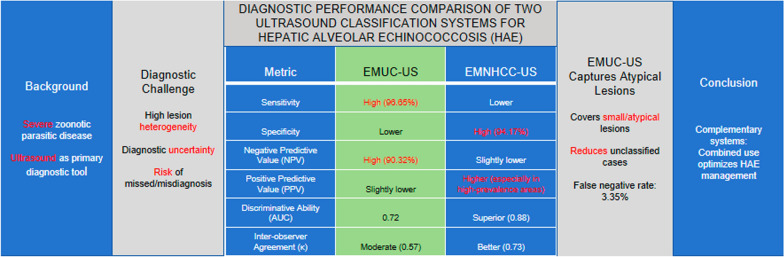

## Background

Alveolar echinococcosis (AE) is a rare zoonotic parasitic disease caused by the larval stage of *Echinococcus multilocularis* in humans [1]. In its life cycle, canids function as definitive hosts, while small mammals such as rodents serve as intermediate hosts. Humans are aberrant hosts and do not contribute to transmission; infection occurs through ingestion of eggs shed in the feces of definitive hosts [2]. AE primarily affects the liver and, similar to cancer, has the potential to invade adjacent tissues or metastasize to distant organs [1]. These hepatic alveolar echinococcosis (HAE) lesions exhibit infiltrative growth with indistinct boundaries between the lesion and surrounding liver tissue. Given that untreated HAE carries a 10-year mortality rate of 90.0% [2, 3], early diagnosis and effective treatment are paramount. China bears the highest global burden of echinococcosis, accounting for approximately 90.0% of worldwide AE cases [1, 3]. A recent national survey reports a 0.2% prevalence of AE in endemic regions, where nearly 60 million residents are at risk. The eastern Qinghai-Tibet Plateau, spanning Sichuan, Qinghai, and Xizang, is a highly endemic area [4]. The significant human health burden of AE, exacerbated by poverty in many endemic communities, highlights the urgent need to strengthen public awareness, surveillance, prevention, and control measures [1]. Improving the diagnosis and management of HAE is a critical public health priority in endemic regions, especially in China.

**Fig. 1:**
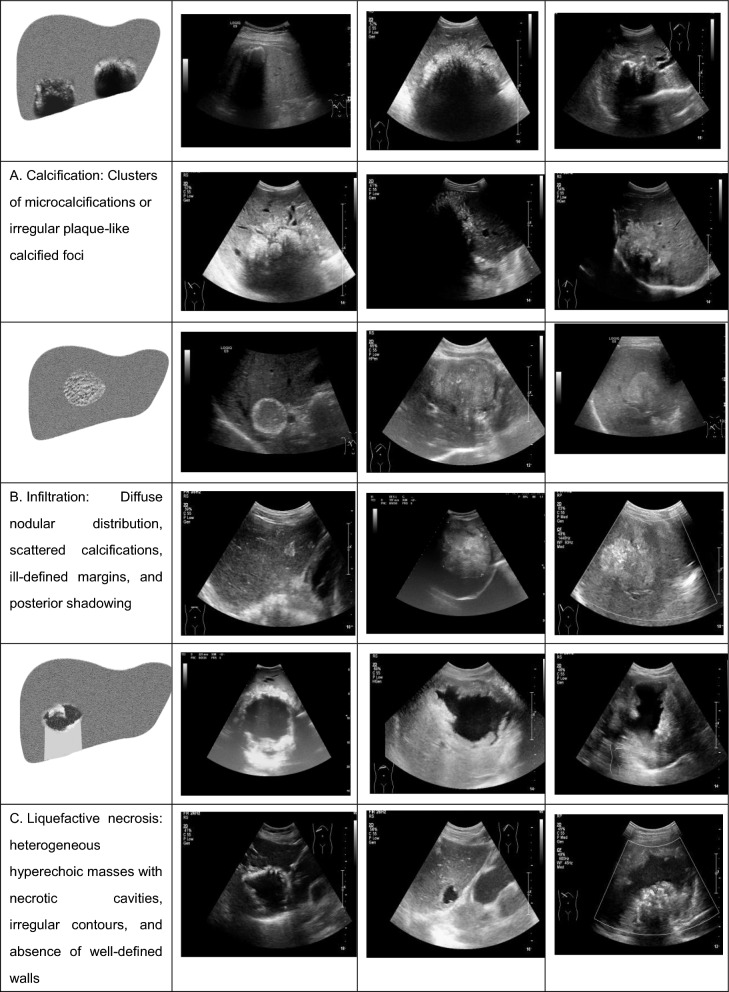
Ultrasound patterns of hepatic alveolar echinococcosis classified according to the *Echinococcus multilocularis* National Health Commission Classification–Ultrasound (EMNHCC-US) system. **A** Calcification: clusters of microcalcifications or irregular plaque-like calcified foci; **B** Infiltration: diffuse nodular distribution with scattered calcifications, ill-defined margins, and posterior acoustic shadowing; **C** Liquefactive necrosis: heterogeneous hyperechoic masses containing necrotic cavities, irregular contours, and no well-defined walls. Representative transabdominal ultrasound images are shown for each pattern

**Fig. 2:**
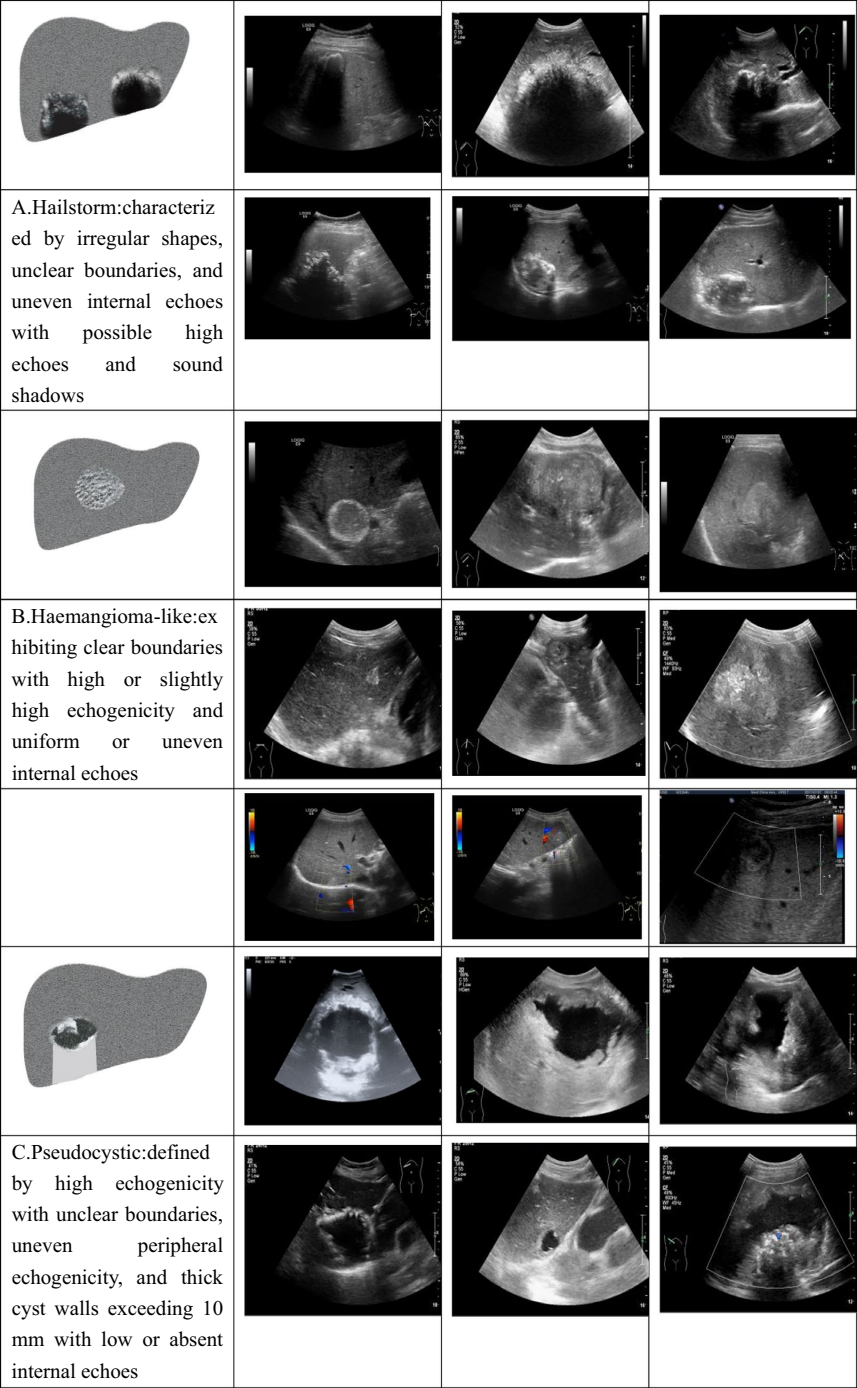

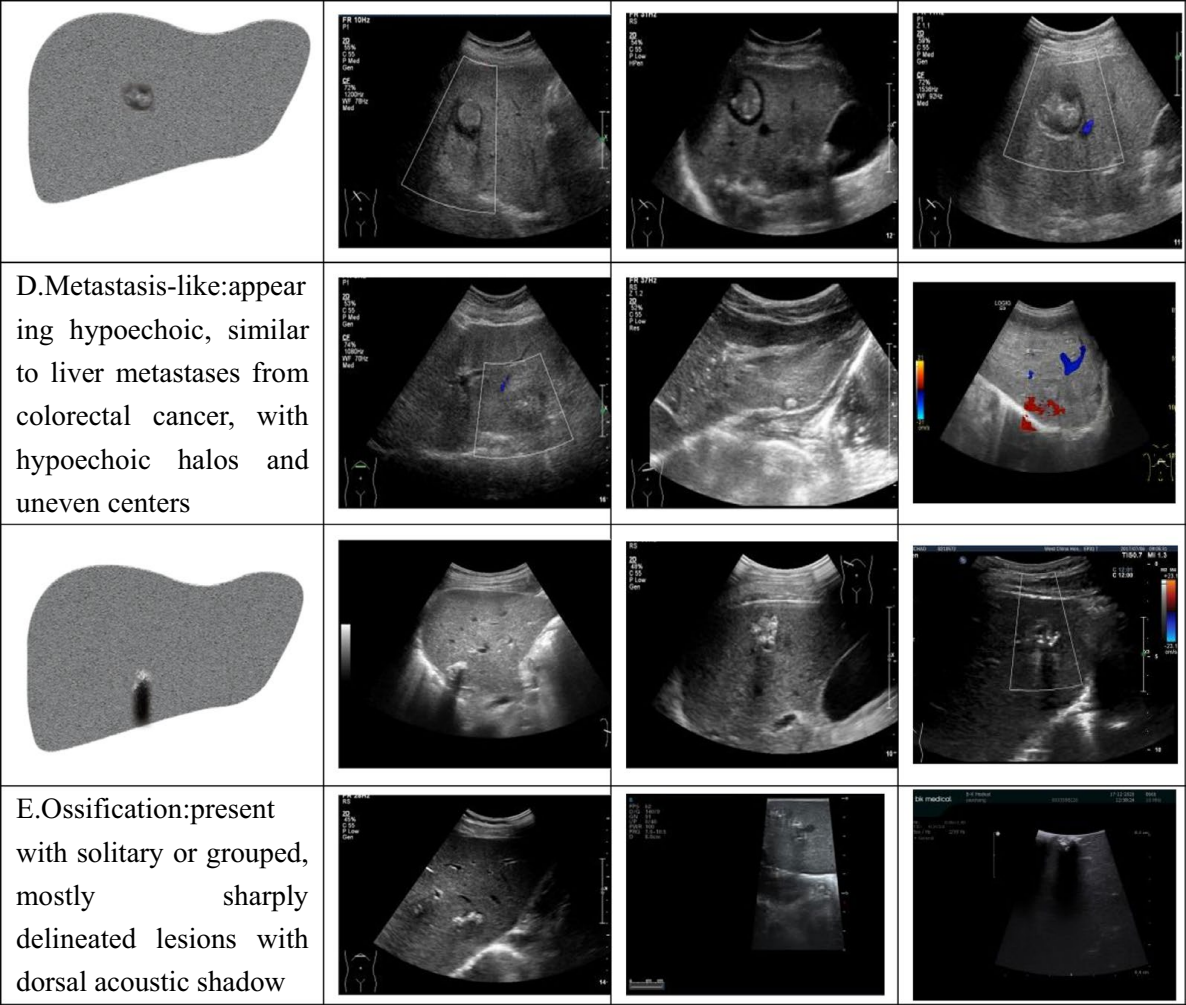
Ultrasound patterns of hepatic alveolar echinococcosis categorized using the *Echinococcus multilocularis* Ulm Classification-Ultrasound (EMUC-US) system. **A** Hailstorm: irregular shape, indistinct margins, heterogeneous internal echoes with hyperechoic foci and posterior shadowing; **B** Hemangioma-like: well-demarcated lesion with high or slightly high echogenicity and uniform or heterogeneous internal echoes; **C** Pseudocystic: hyperechoic rim, unclear borders, peripheral echo heterogeneity, thick cyst wall (> 10 mm), and hypoechoic or anechoic center; **D** Metastasis-like: hypoechoic lesion resembling colorectal liver metastases, often with a hypoechoic halo and heterogeneous central echotexture; **E** Ossification: solitary or clustered lesions with sharp margins and prominent dorsal acoustic shadowing due to calcification or ossification. Representative ultrasound images are provided for each subtype

Ultrasonography is the primary diagnostic tool for detecting HAE [1, 5]. However, the imaging characteristics of HAE lesions are highly heterogeneous, often resemble those of other hepatic pathologies and necessitating careful differential diagnosis [1, 6–8]. Typical morphological features include nodular or irregular shapes with heterogeneous density, strong echogenicity, microcalcifications, and necrotic areas [1, 6–12]. Based on the morphological features of HAE lesions, the WHO Informal Working Group on Echinococcosis proposed three ultrasound-based lesion patterns [6]. These patterns were subsequently adopted by National Health Commission of the People’s Republic of China and formalized into the *E. multilocularis* National Health Commission Classification-Ultrasound (EMNHCC-US) in 2006 (Fig. 1); the EMNHCC-US classification defines three distinct patterns: liquefactive necrosis calcification, and infiltration [7]. In 2015, researchers at Ulm University in Germany introduced an updated ultrasound classification system for HAE lesions, referred to as *E. multilocularis* Ulm Classification-Ultrasound (EMUC-US) (Fig. 2): (1) hailstorm lesions (irregular, unclear boundaries); (2) pseudocystic lesions (thick-walled, hypoechoic centers); (3) hemangioma-like lesions; (4) ossification lesions (acoustic shadowing); and (5) metastasis-like lesions (hypoechoic with halos) [13]. Compared to the EMNHCC-US system, the EMUC-US classification system builds upon it by incorporating two additional patterns, ossification lesions and metastasis-like lesions, potentially improving the characterization of complex HAE presentations to highlight the clinical relevance. The EMNHCC-US classification system has been widely adopted in clinical practice as a major imaging criterion in China for many years [7, 11, 12]. In 2006, the National Health Commission of PRC adopted the EMNHCC-US classification as the national diagnostic criteria for HAE [7]. It has also published several ultrasound images of HAE to demonstrate the three characteristic patterns outlined by the EMNHCC-US system [6]. However, emerging evidence indicates limitations in its capacity to characterize certain HAE lesion morphologies, as demonstrated in a recent evaluation [14]. EMNHCC-US defines three HAE patterns based on pathology, uses non-standardized ultrasound terms, focuses on large lesions, and provides one image per pattern. EMUC-US classifies HAE into five distinct patterns based on standardized sonographic features, including echogenicity, margin, morphology, and size, thereby enhancing reproducibility. The system provides detailed descriptions and multiple representative images, encompassing both early and atypical lesion forms. EMNHCC-US is mainly used in China; EMUC-US in European. Neither has formal diagnostic accuracy evaluation. EMUC-US includes all EMNHCC-US categories and extends to earlier stages, but generalizability across diverse populations requires further validation. Therefore, a comparative analysis of the diagnostic performance of the EMNHCC-US and EMUC-US systems is essential to evaluate their respective clinical utility in diagnosing HAE in China.

## Methods

### Study setting and ethical approval

This retrospective study was conducted at the largest top-tier general hospital in southwestern China serving a province with approximately 90 million residents and over 7.54 million annual visits [15]. The study adhered to the Declaration of Helsinki and was approved by the Ethics Committee of West China Hospital of Sichuan University (Protocol No. 2021WZK1002).

### Case selection

Patients with pathologically confirmed HAE diagnosed between January 2021 and December 2022 were included in the case group. Control subjects were randomly selected from individuals diagnosed during the same period with other hepatic lesions requiring differential diagnosis from HAE [6, 7, 13]. Only patients with high-quality liver ultrasound images available for review were included. Non-HAE lesions included hepatic hemangiomas, primary liver cancer, metastatic liver cancer, hepatic cystic echinococcosis, liver abscess, and hepatic calcifications. Demographic data, including age and sex, were collected through retrospective review of medical records.

### Sonologist selection and grouping

Four senior sonologists with over 10 years of ultrasound experience from tertiary hospitals participated in the diagnostic evaluation. These sonologists neither had prior experience in diagnosing HAE nor received specific training in HAE ultrasound imaging. They were randomly assigned to one of two groups (Group A and Group B), with two sonologists per group. Group A evaluated the lesions using the EMUC-US classification system, while Group B used the EMNHCC-US system. Additionally, two separate senior sonologists, each with over 15 years of experience in HAE diagnosis, were included. Their role was to delineate lesion characteristics and classify all lesions according to the EMUC-US and EMNHCC-US systems. These two experienced sonologists reached consensus on all classifications, serving as the reference standard for the study.

### Questionnaire preparation and data collection

A questionnaire was developed for lesion evaluation (HAE or not) and was distributed via QuestionStar (Zhongyan Technology Co., Ltd., Shanghai, China). It included edited images of 299 lesions from both HAE cases and non-HAE controls. These images were processed using WPS image software (Kingsoft Co., Ltd., Beijing, China) to anonymize patient data and mark lesion locations with arrows. To ensure standardized training, two system-specific slideshows were developed based on the original descriptions and figures of EMUC-US and EMNHCC-US [6, 7, 11, 13]. Both slideshows followed an identical structure, duration (~ 60 min), and instructional format, including definitions, sonographic criteria, and annotated examples. All sonologists received their assigned training in a single session under consistent conditions. Group A was trained with the EMUC-US slideshow, and Group B with the EMNHCC-US slideshow, ensuring equal training duration, content delivery, and learning time for both groups. Following training, both groups received the questionnaire. Each group was provided with a reference manual containing images and lesion descriptions corresponding to their assigned classification system: Group A received the EMUC-US manual, while Group B received the EMNHCC-US manual. These references remained accessible during evaluation to guide assessments. Finally, Group A assessed lesions using the EMUC-US system, while Group B employed the EMNHCC-US system. Neither group had prior knowledge of the pathological diagnoses.

### Statistical analysis

Statistical analyses were conducted using R software (R Foundation for Statistical Computing, Vienna, Austria). Descriptive statistics (means, proportions) were calculated. Between-group comparisons of continuous variables (means) and categorical variables (proportions) were assessed using appropriate parametric or non-parametric tests. Inter-observer reliability within each sonologist group was evaluated using Cohen’s Kappa statistic. κ values indicate slight (< 0.20), fair (0.21–0.40), moderate (0.41–0.60), substantial (0.61–0.80), or almost perfect (0.81–1.00) agreement [16, 17]. Sensitivity, specificity, positive predictive value (PPV), and negative predictive value (NPV) were calculated for each classification system. A receiver operating characteristic (ROC) curve was generated for each system using pROC package, and the area under the curve (AUC) along with its 95.0% confidence interval (*CI*) was computed. Statistical significance was set at *P* < 0.05. A sensitivity analysis was performed to assess the impact of disease prevalence on diagnostic performance. PPV and NPV were recalculated across prevalence rates of 0.001%, 0.01%, 0.1%, 1.0%, 2.5%, and 5.0% using Bayes’ theorem.

## Results

### General information

The study included 169 cases with pathologically confirmed HAE in the case group, with a mean age of 37.46 ± 1.86 years. Among these patients, 87 (51.5%) were male and 82 (48.5%) were female, with a total of 179 hepatic lesions identified. The control group consisted of 99 non-HAE patients with a mean age of 46.02 ± 2.51 years, including 55 (55.6%) males and 44 (44.4%) females, who collectively presented with 120 lesions. HAE lesions predominantly affected segments of the right hepatic lobe (Tables 1, 2). Ultrasound evaluation of all 179 HAE lesions revealed universal heterogeneous echotexture (100.0%). Specific sonographic characteristics included hypoechoic appearance in 55.9% (100/179), anechoic areas in 44.7% (80/179), hyperechogenicity with acoustic shadowing in 38.0% (68/179), hyperechogenicity without acoustic shadowing in 32.4% (58/179), and anechoic areas with posterior acoustic enhancement in 29.6% (53/179). In this study, the EMUC-US system identified pseudocystic lesions as the most prevalent pattern (36.3%), followed by haemangioma-like (25.7%) and hailstorm (22.9%) lesions. The EMNHCC-US system classified infiltration lesions as the most common (49.7%), followed by liquefactive necrosis (31.8%).

**Table 1:** General information of HAE cases and lesion characteristics classified using the EMUC-US

Lesion pattern	No. lesions	Proportion (%)	Lesion size (mean max-diameter ± *SD*, cm)	Lesion location within liver, *n* (%)		
				Left liver	Right liver	Left and right liver
Pseudocystic	65	36.3	12.13 ± 1.12	4 (6.2)	21 (32.3)	40 (61.5)
Haemangioma-like	46	25.7	7.37 ± 1.25	7 (15.2)	25 (54.4)	14 (30.4)
Hailstorm	33	18.4	8.95 ± 1.35	0 (0.0)	15 (45.5)	18 (54.5)
Metastatic-like	19	10.6	3.17 ± 1.29	8 (42.1)	9 (47.4)	2 (10.5)
Ossified	11	6.1	0.96 ± 0.29	4 (36.4)	7 (63.6)	0 (0.0)
Unclassified	5	2.8	5.66 ± 4.06	2 (40.0)	1 (20.0)	2 (40.0)
Total	179	100.0	8.51 ± 0.74	25 (14.0)	78 (43.6)	76 (42.5)

*EMUC-US Echinococcus multilocularis* Ulm Classification-Ultrasound, *HAE* hepatic alveolar echinococcosis, *SD* Standard deviation

**Table 2:** General information of HAE cases and lesion characteristics classified using classified using the EMNHCC-US

Lesion pattern	No. lesions	Proportion (%)	No. lesions	Lesion size (mean max-diameter ± SD, cm)	Lesion location within liver, *n* (%)		
					Left liver	Right liver	Left and right liver
infiltration	89	49.7	89	7.59 ± 0.82	16 (18.0)	41 (46.1)	32 (36.0)
liquefactive necrosis	57	31.8	57	12.50 ± 1.08	4 (7.0)	16 (28.1)	37 (64.9)
calcification	15	8.4	15	8.34 ± 1.86	0 (0.0)	9 (60.0)	6 (40.0)
Unclassified	18	10.1	18	2.61 ± 2.61	5 (27.8)	12 (66.7)	1 (5.6)
Total	179	100.0	179	8.51 ± 0.74	25 (14.0)	78 (43.6)	76 (42.5)

*EMUC-US Echinococcus multilocularis* Ulm Classification-Ultrasound, *HAE* hepatic alveolar echinococcosis, *SD* Standard deviation

### Consistency analysis of two classification systems

Inter-observer agreement between sonologists was assessed using κ for both ultrasound classification systems. The EMUC-US system demonstrated moderate inter-observer consistency with a κ value of 0.57 (95% *CI:* 0.47–0.67), while the EMNHCC-US system showed substantially higher agreement with a κ value of 0.73 (95% *CI:* 0.65–0.81) (Table 3). Statistical analysis confirmed that both κ values were significantly different from zero (*P* < 0.05), indicating reliable performance of both classification systems.

**Table 3:** Consistency analysis of diagnostic results: Inter-observer agreement

	κ value	Standard error	Test statistic	*P* value
EMNHCC-US	0.73	0.04	12.603	< 0.05
EMUC-US	0.57	0.05	9.99	< 0.05

*EMNHCC-US Echinococcus multilocularis* National Health Commission Classification-Ultrasound, *EMUC-US Echinococcus multilocularis* Ulm Classification-Ultrasound

### Diagnostic performance

Comparison of diagnostic performance between the EMUC-US and EMNHCC-US systems revealed statistically significant differences across all measured parameters (*P* < 0.05). The EMUC-US system showed sensitivity of 96.7%, specificity of 46.7%, PPV of 73.0%, NPV of 90.3%, and an overall accuracy of 76.6%. In contrast, the EMNHCC-US system demonstrated sensitivity of 82.7%, specificity of 94.2%, PPV of 95.5%, NPV of 78.5%, and an overall accuracy of 87.3%. Statistical analysis using chi-square tests confirmed significant differences between the systems for sensitivity (*χ*^2^ = 17.36, *P* < 0.05), specificity (*χ*^2^ = 62.73, *P* < 0.05), PPV (*χ*^2^ = 30.45, *P* < 0.05), NPV (*χ*^2^ = 6.37, *P* < 0.05), and overall accuracy (*χ*^2^ = 10.86, *P* < 0.05) (Table 4).

**Table 4:** Comparison of diagnostic performance indicators between the EMUC-US and EMNHCC-US systems

Diagnostic indicator	EMNHCC-US (%)	EMUC-US (%)	*χ*^2^	*P* value
Sensitivity	82.7	96.7	17.36	< 0.05
Specificity	94.2	46.7	62.73	< 0.05
Positive predictive value	95.5	73.0	30.45	< 0.05
Negative predictive value	78.5	90.3	6.37	< 0.05
Accuracy	87.3	76.6	10.86	< 0.05

*EMNHCC-US Echinococcus multilocularis* National Health Commission Classification-Ultrasound, *EMUC-US Echinococcus multilocularis* Ulm Classification-Ultrasound

### ROC curve analysis

**Fig. 3:**
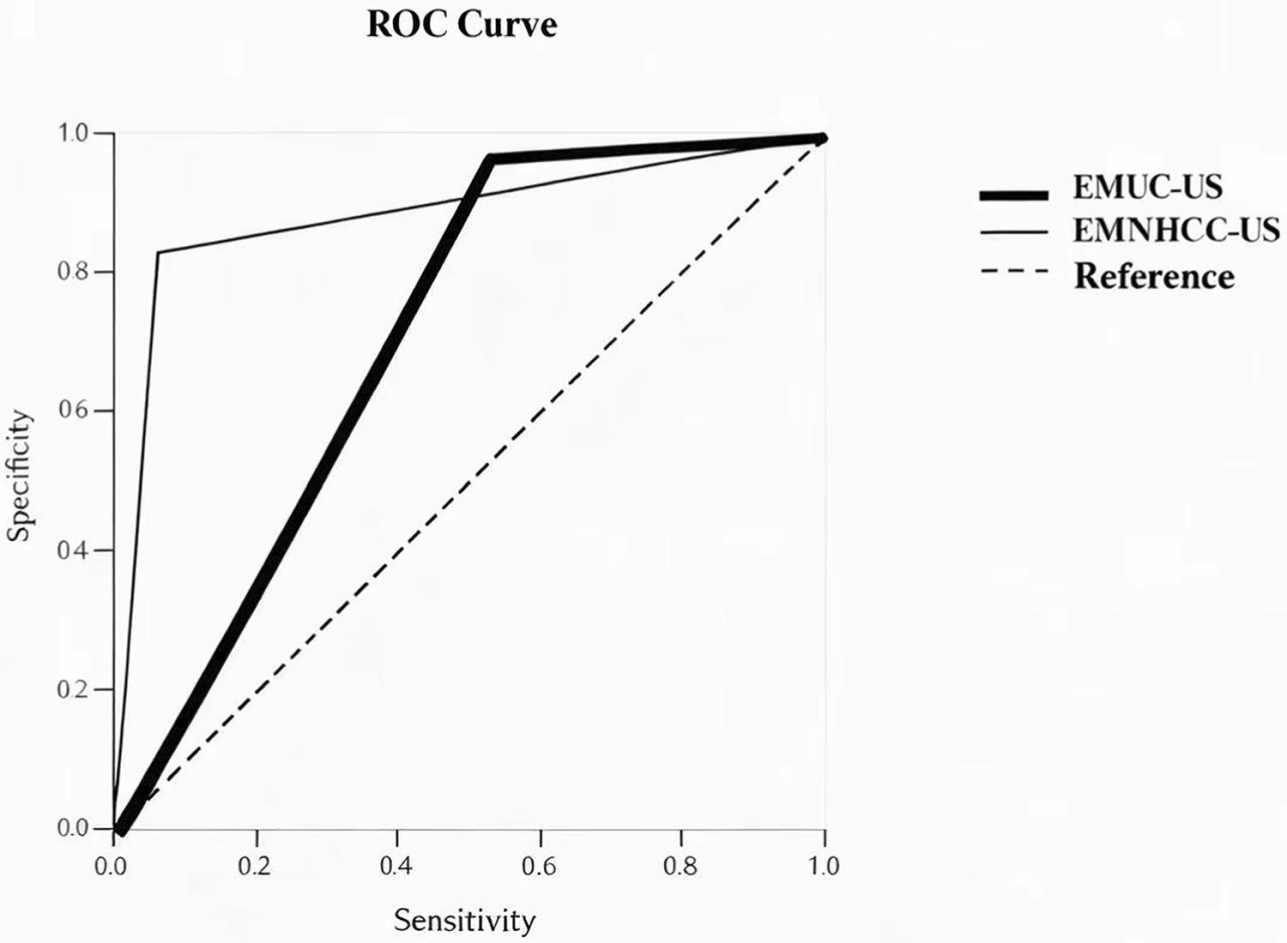
Receiver operating characteristic (ROC) curves comparing the diagnostic performance of the *Echinococcus multiloculari*s Ulm Classification-Ultrasound (EMUC-US) and the *Echinococcus multilocularis* National Health Commission Classification-Ultrasound (EMNHCC-US) systems in differentiating hepatic alveolar echinococcosis (HAE) from other liver lesions. The area under the curve (AUC) quantifies each system's discriminative accuracy, with higher values indicating better diagnostic performance

ROC curve analysis (Fig. 3) demonstrated that the EMNHCC-US system exhibited better discriminative performance compared to the EMUC-US system. The EMNHCC-US system achieved an AUC of 0.88 (95% *CI:* 0.84–0.93), whereas the EMUC-US system had an AUC of 0.72 (95% *CI:* 0.65–0.78).

### Sensitivity analysis

EMUC-US has a low false negative rate (FNR, 3.4%) and near-perfect NPV (~ 100.0%) across all prevalence rates, suggesting a minimal risk of missed diagnoses. EMNHCC-US has a higher FNR (17.3%) but better PPV at high prevalence (e.g., 42.7% vs. 8.7% at 5.0% prevalence rate), consistent with its higher specificity. EMUC-US’s PPV remains low at low prevalence rates because of its lower specificity (Table 5).

**Table 5:** Sensitivity analysis of the EMUC-US and EMNHCC-US classification systems at varying prevalence levels

Prevalence (%)	EMUC-US		EMNHCC-US	
	PPV (%)	NPV (%)	PPV (%)	NPV (%)
0.001	0.002	100.000	0.014	100.000
0.01	0.018	99.999	0.142	99.998
0.10	0.181	99.993	1.400	99.982
1.0	1.798	99.928	12.530	99.815
2.5	4.441	99.816	26.667	99.531
5.0	8.708	99.624	42.740	99.041

*EMNHCC-US Echinococcus multilocularis* National Health Commission Classification-Ultrasound, *EMUC-US Echinococcus multilocularis* Ulm Classification-Ultrasound, *PPV* Positive predictive value, *NPV* Negative predictive value

## Discussion

HAE requires early ultrasound diagnosis [18–22], yet its low prevalence limits sonographer experience [23–25]. While two ultrasound classification systems for HAE have been proposed [7, 13, 26, 27], their comparative performance was unestablished. To address this, we conducted a blinded review of the EMNHCC-US and EMUC-US systems. All 179 HAE lesions exhibited heterogeneous echotexture (100.0%). Key features were hypoechogenicity (55.9%), anechoic areas (44.7%), hyperechogenicity with shadowing (38.0%), without shadowing (32.4%), and posterior enhancement (29.6%). The co-occurrence of these features (> 100.0% cumulative) highlights extreme pathological heterogeneity [1, 13, 20, 25]. This stems from *E. multilocularis*'s infiltrative growth, invading liver parenchyma without a capsule and inciting a peripheral granulomatous response with fibrosis, necrosis, and microvesicles [5, 6, 20]. This pathological complexity directly drives the complicated and overlapping sonographic presentations [1, 13, 20, 25].

EMUC-US and EMNHCC-US demonstrated distinct performance profiles with important clinical implications. EMUC-US showed high sensitivity (96.7%) and negative predictive value (90.3%), maintaining near-perfect NPV across prevalence levels, supporting its use as a screening tool to rule out HAE in endemic areas. In contrast, EMNHCC-US achieved higher specificity (94.2%) and superior discriminative ability (AUC: 0.88 vs. 0.72), making it more suitable for confirming advanced disease. However, neither system reached the AUC > 0.90 threshold for high diagnostic accuracy, underscoring the need for refinement. EMUC-US also enabled detection of smaller, atypical lesions (e.g., ossification, metastasis-like), expanding early case identification. Despite acceptable inter-observer agreement (κ = 0.57–0.73) [16, 17], both systems have limitations in real-world settings, particularly given HAE's low incidence and asymptomatic early phase [1, 2]. The lower PPV of EMUC-US at low prevalence necessitates confirmatory testing for positive findings, while EMNHCC-US may miss early lesions due to lower sensitivity (82.7%). These findings highlight a critical gap: current classifications lack sufficient accuracy for standalone diagnosis. Refinement—through standardized criteria, broader validation, and integration of imaging advances—is essential to improve early detection and diagnostic certainty in global HAE management [16, 17, 28, 29].

The proportion of lesion patterns observed in this study differed from those described in the original EMUC-US classification, which was proposed by doctors from a German hospital [13]. Notably, the proportion of pseudocystic lesions was higher in our study. The original report from the German hospital described the following lesion composition: 25 pseudocystic lesions (13.5%), 15 haemangioma-like lesions (8.1%), 100 hailstorm lesions (54.1%), 24 ossified lesions (13.0%), 12 metastatic-like lesions (6.5%), and 9 unclassified lesions (4.9%) [13]. In contrast, our study found pseudocystic lesions accounted for 36.3% (65,179), and haemangioma-like lesions for 25.7% (46/179), both significantly higher than the German study (*P* < 0.05). While our study encompassed all lesion types described in the EMUC-US classification, a previous smaller study (58 lesions) did not identify any metastatic-like patterns [18]. The absence of metastatic-like lesions in the earlier study further underscores the importance of validating classification systems across diverse and larger populations to ensure broad applicability in clinical practice.

Several limitations may influence the validity and generalizability of these findings. First, the retrospective, single-center design limits external applicability, particularly to primary care or low-resource settings where HAE is often first suspected. Selection bias may arise from concentrating on confirmed cases at a tertiary referral center, potentially overrepresenting advanced or atypical presentations and underestimating the prevalence of early-stage, subtle lesions in the general population. Second, the sonologists, though uniformly trained, lacked prior experience in HAE diagnosis. While this standardization reduces variability in baseline knowledge, it may underestimate the performance achievable by experienced operators and overestimate the ease of system adoption in real-world clinical settings. This artificial homogenization of expertise could bias performance metrics upward or downward depending on case complexity, particularly for ambiguous or small lesions. Third, assessment was limited to static ultrasound images, excluding real-time dynamic evaluation, such as vascular flow patterns on Doppler or mobility during transducer pressure, which are clinically relevant for differential diagnosis. The absence of these dynamic cues likely reduced diagnostic confidence and accuracy, particularly in distinguishing HAE from malignancies or hemangiomas, thereby potentially underestimating the true potential of either system when applied in routine practice.

The considerable diversity in grayscale ultrasound manifestations of HAE lesions, demonstrated in this study, highlights the critical need to systematically characterize these features [1, 13, 25, 27–29]. This is a foundational step for advancing imaging research and implementing One Health-aligned precision screening (focusing on human health data integration). While our classification system comparison offers valuable insights, multicenter validation remains essential for robust performance assessment. Diagnosing small lesions, particularly ossification and metastasis-like patterns that require differentiation from other hepatic pathologies [1, 20], presents significant challenges [28–30].

## Conclusions

EMUC-US and EMNHCC-US play complementary roles: EMUC-US, with its broader categorization of early and atypical lesions, offers high sensitivity ideal for screening, while EMNHCC-US provides high specificity for confirming advanced disease. Their combined use supports a stratified diagnostic approach—ruling out HAE with EMUC-US and confirming it with EMNHCC-US. Despite their value, neither system achieves optimal accuracy, highlighting the need for refinement. Multicenter validation across diverse populations is essential to enhance generalizability. Future efforts should integrate advanced technologies—such as artificial intelligence, contrast-enhanced ultrasound, and machine learning—and align with a One Health approach, bridging human, animal, and environmental surveillance to improve early detection and control of this complex zoonotic disease.

## Data Availability

Data is available at the Department of Medical Ultrasound, West China Hospital, Sichuan University, Chengdu 610041, Sichuan Province, China and fully accessible to all co-authors. Data can be shared with other institutions and researchers upon request.

## Abbreviations

AUC: Area under curve of receiver operating characteristic
EMNHCC-US: *Echinococcus multilocularis* National Health Commission Classification on Echinococcosis system-Ultrasound
EMUC-US: *Echinococcus multilocularis* Ulm Classification-Ultrasound
HAE: Hepatic alveolar echinococcosis
PPV: Positive predictive value
NPV: Negative predictive value
AUC: Area under the curve of receiver operating characteristic
ROC: Receiver operating characteristic
WHO: World Health Organization
SD: Standard deviation
*CI*: confidence interval
AE: Alveolar echinococcosis
